# Geospatial and Cell Density Analysis Using Multiplex Immunofluorescence Reveals an Important Role of Clustering Patterns of Immunosuppressive Macrophages in Survival Outcomes of Penile Squamous Cell Carcinoma

**DOI:** 10.3390/cancers18020257

**Published:** 2026-01-14

**Authors:** Adnan Fazili, Keerthi Gullapalli, Gabriel Roman Souza, Firas Hatoum, Justin Miller, Youngchul Kim, Junmin Whiting, Jeffrey S. Johnson, Jasreman Dhillon, Jonathan Nguygen, Carlos Moran Segura, Philippe E. Spiess, Jad Chahoud

**Affiliations:** 1 Department of Genitourinary Oncology, H. Lee Moffitt Cancer Center, Tampa, FL 33612, USA; adnan.fazili@moffitt.org (A.F.); keerthi.gullapalli@moffitt.org (K.G.); gabriel.romansouza@moffitt.org (G.R.S.); firas.hatoum@moffitt.org (F.H.); jeff.johnson@moffitt.org (J.S.J.); philippe.spiess@moffitt.org (P.E.S.); 2 Department of Medicine, USF Health Morsani College of Medicine, Tampa, FL 33612, USA; justin.w.miller@moffitt.org; 3 Department of Biostatistics, H. Lee Moffitt Cancer Center, Tampa, FL 33612, USA; youngchul.kim@moffitt.org (Y.K.); junmin.whiting@moffitt.org (J.W.); jonathan.nguyen@moffitt.org (J.N.); carlos.moransegura@moffitt.org (C.M.S.); 4 Department of Pathology, H. Lee Moffitt Cancer Center, Tampa, FL 33612, USA; jasreman.dhillon@moffitt.org; 5 Orlando Health Cancer Institute, Orlando, FL 32806, USA

**Keywords:** penile squamous cell carcinoma, tumor immune microenvironment, multiplex immunofluorescence, human papillomavirus, spatial profiling, tumor-associated macrophages, prognosis

## Abstract

Background/Objectives: Penile squamous cell carcinoma (PSCC) is a rare malignancy with poor prognosis in advanced and recurrent disease, and therapeutic options remain limited. Increasing evidence suggests that the tumor immune microenvironment (TIME), including immune cell composition and spatial organization, plays a critical role in tumor progression and survival outcomes. This study aimed to characterize immune cell density and geospatial clustering patterns within the TIME of PSCC and to evaluate their associations with clinical outcomes. Methods: Multiplex immunofluorescence (mIF) was performed on tumor samples from 57 patients with PSCC using a panel of immune markers to identify lymphoid and myeloid cell populations. Immune cell densities were quantified within tumoral and stromal compartments. Spatial relationships among immune cells and between immune cells and tumor cells were analyzed using point pattern analysis. Survival outcomes, including overall survival (OS), recurrence-free survival (RFS), and cancer-specific survival (CSS), were assessed using Kaplan–Meier methods and Cox proportional hazards models, with analyses stratified by nodal and human papillomavirus (HPV) status. Results: Higher intratumoral and stromal densities of pro-immunogenic M1 macrophages were associated with improved OS. Increased densities of CD3^+^CD4^+^ helper T cells in both compartments were also associated with favorable survival outcomes. In contrast, close clustering of pro-tumorigenic M2 macrophages with tumor cells and with one another was associated with worse OS, RFS, and CSS. Bivariate clustering of helper T cells with tumor cells was associated with improved OS, including among patients with node-positive disease. Survival outcomes did not differ significantly by HPV status in patients with high helper T cell clustering. Conclusions: Immune cell density and spatial organization within the TIME are associated with survival outcomes in PSCC. Favorable patterns involving helper T cells and M1 macrophages correlate with improved survival, whereas clustering of M2 macrophages is associated with poorer outcomes, supporting the relevance of spatial immune profiling in this disease.

## Simple Summary

Penile cancer is a rare malignancy with poor prognosis in advanced and recurrent stages. The currently limited treatment options and poor survival outcomes represent an unmet need for patients with advanced or recurrent penile cancer. Innovation in therapeutic options for patients with penile cancer relies on insight of underlying tumor biology and tumor–immune system interactions. In this paper, we use multiplex immunofluorescence to investigate the immune microenvironment of penile cancer, focusing specifically on geospatial clustering patterns of various immune effector cells within various stages of penile cancer and human papillomavirus status. We identified spatial and clustering patterns of pro-immunogenic macrophages, tumor-associated macrophages, and helper T cells as potential prognosticators of survival in penile squamous cell carcinoma.

## 1. Introduction

Penile cancer is a rare malignancy that is both psychologically overwhelming for patients and clinically challenging to manage. Advanced stages of penile cancer have a poor prognosis and are often fatal. Approximately 2100 new cases of penile cancer are estimated in the United States in 2024 with about 500 expected deaths [1]. Penile cancer accounts for fewer than 1% of cancers in men in the United States; however, it is much more common in some parts of Asia, Africa, and South America, where the incidence can account for up to 10% of malignancies [2,3]. Most penile cancers are of squamous cell origin with frequent association with the human papillomavirus (HPV) [4]. Other risk factors associated with penile cancer include tobacco use, phimosis, poor hygiene, and low socioeconomic status [5].

Localized penile cancer can be treated with topical therapy, radiation, organ-sparing, and radical surgical techniques [6,7,8]. Management of advanced penile cancer relies on accurate assessment of regional lymph nodes because patients with small-volume regional lymph nodes can be treated with primary curative surgery, while those with bulky lymph node involvement are thought to benefit from neoadjuvant therapy followed by surgical consolidation [9,10]. The optimal sequence of treatment in locoregional and advanced stages of penile cancer is a topic of extensive study, largely due to a lack of a single efficacious treatment modality. The prognosis and survival in locoregional and metastatic disease remain poor, regardless of treatment sequence. While 50% of patients achieve an objective response to systemic therapy, the majority of patients will have disease recurrence and poor long-term survival [11,12,13].

For patients with advanced disease, there is a need to develop more effective treatment strategies. Immune checkpoint inhibitors and monoclonal antibodies have revolutionized the management of many squamous cell cancers (SCC) [14]. Recent studies on immunotherapy have shown potential benefits in advanced penile cancer, although responses to programmed cell death protein 1 (PD-1) and programmed death-ligand 1 (PD-L1) immune checkpoint inhibitors remain limited among patient populations [15,16]. Identifying predictive biomarkers may help select certain patients for therapy escalation [17].

The tumor immune microenvironment (TIME) describes a complex ecosystem that plays a crucial role in cancer progression. Gaining a thorough understanding of the TIME can improve patient stratification by prognosis, reveal new immunotherapy targets, and help predict responses to currently approved immune-based treatments. Multiplex immunofluorescence (mIF) has emerged as an important tool for profiling the TIME. Previous studies using mIF have described a pattern of immune exhaustion with advancing stages of penile squamous cell carcinoma (PSCC) and highlighted the role of myeloid cells in creating an immunotolerant, pro-tumorigenic environment [18]. Single cell transcriptomic efforts have shown an increase in pro-tumorigenic M2 macrophages and exhausted T cells in advanced stages of PSCC.

In this study, we investigated the cellular density pattern and spatial interactions of helper T cells, M2 macrophages, and other immune populations within the TIME in relation to survival outcomes across varying stages and viral profiles of PSCC [19]. We hypothesize that close clustering patterns of M2 macrophages within the tumor compartment could be associated with poorer survival outcomes, whereas increased infiltrating lymphocytes within the tumor compartments would be associated with improved survival outcomes.

## 2. Materials and Methods

### 2.1. Multiplex Immunofluorescence and Image Analysis

A tissue microarray (TMA) was developed using formalin-fixed, paraffin-embedded (FFPE) samples from 57 retrospective cases of invasive PSCC. All tissue samples were obtained from primary tumor resections. mIF was conducted to evaluate immune cell populations, employing a panel of ten immune markers: CD20, CD3, CD4, CD8, CD45RO, CD68, CD206, CD163, NKp46, and FOXP3. Staining was performed using the OPAL™ 7-Color Automation IHC Kit (AKOYA Biosciences, Waltham, MA, USA) on the BOND RX autostainer (Leica Biosystems, Vista, CA, USA) [20]. These markers were selected based on prior evidence linking their expression patterns to response to immunotherapy across multiple solid tumors and their established relevance to the TIME of PSCC.

Standard protocols for incubation, deparaffinization, and antigen retrieval—previously described in detail—were followed [18]. Negative controls included slides processed without OPAL fluorophores or DAPI, as well as omission of primary or secondary antibodies. All stained slides were scanned using the Vectra^®^3 Automated Quantitative Pathology Imaging System. Image data were processed in InForm 3.0.0 (Akoya) and analyzed using the HALO Image Analysis Platform 4.1 (Indica Labs, Albuquerque, NM, USA).

### 2.2. Cell Density Analysis, Phenotyping and Compartmentalization

Cytokeratin (CK) was used as a marker to divide the intratumoral region into two distinct compartments: the tumor compartment, consisting of clusters of malignant cells, and the stromal compartment, comprising the fibrous tissue situated between these tumor clusters. A supervised machine learning algorithm, based on random forest classification, was trained to distinguish between tumor and stromal regions for quantitative analysis. The density of each identified cell phenotype was quantified and expressed as the number of cells per square millimeter (cells/mm^2^). All data analyses were conducted using RStudio version 3.5.3, utilizing the Phenoptr 0.2.2 package from Akoya Biosciences.

Chord plots were created to visualize interactions among cell phenotypes based on the co-expression of markers using the markers from each mIF panel [21].

### 2.3. Geospatial Analysis

The spatial arrangement of cell phenotypes was analyzed using point pattern analysis, focusing on immune cell clustering within a 50 nanometers (nm) radius. Positive values indicated clustering, while negative values suggested dispersion. Additionally, distances between immune and malignant cells were calculated using their x- and y-coordinates in RStudio version 4.2.3. This approach aligns with established methods for assessing spatial relationships in tumor microenvironments.

For geospatial analysis of mIF data, immune cell clustering was evaluated using a normalized univariate Ripley’s K function at a defined search radius of 50 nm. Positive values indicated a tendency toward spatial clustering, while negative values reflected cellular dispersion.

Univariate clustering assessed the spatial organization of cells of the same phenotype (e.g., whether cell type A clusters with other type A cells), whereas bivariate clustering examined spatial relationships between different cell types (e.g., the tendency of cell type A to cluster with cell type B).

Programmed death-ligand 1 (PD-L1) expression was evaluated at the TMA level and quantified using the Quickscore method [22]. All samples positive for p16 IHC underwent testing for high-risk HPV. FFPE tissue sections from PSCC were processed and pretreated according to the Cobas^®^ HPV assay (Roche Diagnostics, Indianapolis, IN, USA) [23,24].

### 2.4. Statistical Analysis

Statistical comparisons of quantitative variables between groups were performed using the Kruskal–Wallis test. Categorical variables were analyzed using either the Chi-square test or Fisher’s exact test, as appropriate.

Survival outcomes—including recurrence-free survival (RFS), overall survival (OS), and cancer-specific survival (CSS)—were measured from the time of diagnosis to recurrence, death, or last follow-up. Kaplan–Meier survival curves and log-rank tests were used to assess associations between survival outcomes and dichotomized mIF-derived metrics (high versus low), determined using the Maximally Selected Rank Statistics (MaxStat) method. Cox proportional hazards models were then employed to analyze both continuous and dichotomized predictors of survival.

## 3. Results

### 3.1. Study Population

A total of 57 male patients with a confirmed diagnosis of PSCC were retrospectively included in this study between 2010 and 2016. The median age of the patients was 60, ranging from 31 to 92 years. The majority of patients were Caucasian, reflecting the demographics of the institutional catchment area. HPV positivity was found in 40% of tumors. Primary resection included partial penectomy (70%), total penectomy (21%), wide local excision (7%), and circumcision (2%). All patients either had clinically node-positive disease or high-risk primary penile cancer, and all underwent an inguinal lymph node dissection (ILND). Thirty-one of fifty-seven (54%) patients had pathologically node-positive disease at ILND. Of the 26 patients with node-negative disease (pN0), 81% had intermediate- or high-risk primary disease, defined as ≥pT1b. A total of 30% of patients received adjuvant therapy which included chemoradiotherapy (47%), cisplatin-based combination chemotherapy alone (35%), and radiotherapy alone (18%).

Pathologic examination revealed lymph node involvement in 31 of 57 patients (54%). Among those without nodal involvement, 21 of 26 (81%) had intermediate- or high-risk primary disease (T1 with grade 3 or ≥T2). Nearly one-third (17 out of 57, or 30%) received adjuvant therapy: six received cisplatin-based chemotherapy alone, three underwent radiotherapy alone, and eight received a combination of chemoradiotherapy. Baseline clinicopathologic features are presented in Table 1.

### 3.2. Survival Outcomes and Prognostic Factors

Overall, 45.6% of patients experienced a recurrence during follow-up. The majority of recurrences were inguino-pelvic recurrences, followed by distant metastatic disease. Only five local recurrences were seen. The median time to recurrence was 6.3 months (IQR 3.1–12.7). The median OS was 36.3 months (IQR 18.9–52.31) and the median follow up was 45.5 months (IQR 29.3–93.6). Over half of patients died during follow-up, and among those who died, over a third of patients were dead from disease. Survival and oncological outcomes are delineated in Table 2. Table 3 illustrates univariate cox proportional hazards model for overall survival. HPV-negative status and locoregionally advanced disease (≥pN2) were associated with a decreased likelihood of survival. PD-L1 status, receipt of adjuvant therapy, primary tumor stage, and presence of lymphovascular invasion (LVI) were not associated with a decreased likelihood of survival.

### 3.3. Immune Phenotypes in mIF Panels

Table 4 describes the phenotypic distribution of various immune effector cells across tumoral and stromal compartments. Median cell densities were higher within stromal compartments across all cell types. The chord plot demonstrated distinct patterns of cellular phenotypes and cell–cell interactions within the overall cohort. A relatively low number of cytotoxic T lymphocytes (CD3^+^CD8^+^) and helper T lymphocytes (CD3^+^CD4^+^) were observed compared with memory T cells (CD45RO^+^). The median cell density of M1 macrophages (CD68^+^CD163^−^CD206^−^) exceeded the total median cell density of M2 macrophages (CD68^+^CD163^+^, CD68^+^CD206^+^).

### 3.4. High Clustering of M2 Macrophages to One Another and Tumor Cells Is Associated with Worse Overall Survival

**Figure 1:**
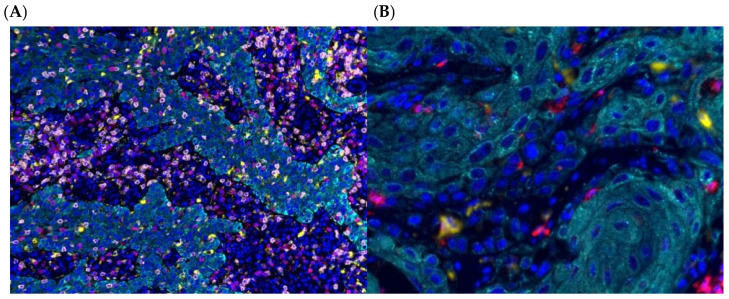
mIF illustration of high versus low cell density and clustering of M2 macrophages. Representative multiplex immunofluorescence (mIF) images illustrating high (**A**) versus low (**B**) M2 macrophage cell density and spatial clustering within the tumor microenvironment.

**Figure 2:**
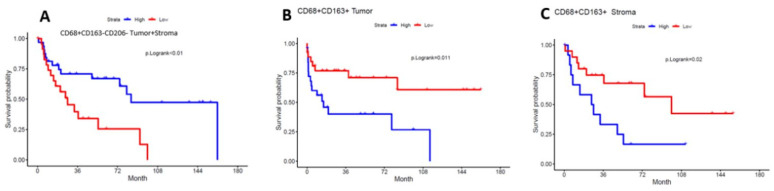
Overall survival (OS) according to macrophage density and spatial clustering patterns. (**A**) Kaplan–Meier analysis of OS stratified by high versus low density of M1 macrophages within the intratumoral compartment. (**B**) Kaplan–Meier analysis of OS based on bivariate intratumoral clustering between M2 macrophages and tumor cells. (**C**) Kaplan–Meier analysis of OS according to univariate stromal clustering of M2 macrophages. High and low groups were defined using maximally selected rank statistics.

Figure 1 shows a visual illustration of high versus low cell density and clustering patterns of tumor-associated macrophages within two different patient tumor specimens. High versus low intratumoral and stromal cell density of M1 macrophages (CD68^+^CD163^−^CD206^−^) was associated with improved overall survival; 91 versus 28 months, *p* < 0.005 (Figure 2A). Within the intratumoral compartment, close clustering of M2 macrophages (CD68^+^CD163^+^) with tumor cells was associated with worse overall survival 19 months vs. not reached, *p* < 0.011 (Figure 2B). The close co-clustering of M2 macrophages to one another within the stromal compartment portends a worse median OS 28 vs. 96 months, *p* = 0.02 (Figure 2C). Moreover, high clustering of M2 macrophages to tumor cells was also associated with worse RFS: 9 months versus not reached, *p* = 0.013. HR 2.94 95% CI (1.24–6.96), *p* = 0.014, and worse CSS: 26 months versus not reached, *p* = 0.03. HR 3.77 95% CI (1.2–11.3), *p* = 0.018.

### 3.5. High Cell Density and Geospatial Clustering of Tumor-Infiltrating Helper T Cells Is Associated with Improved Overall Survival

**Figure 3:**
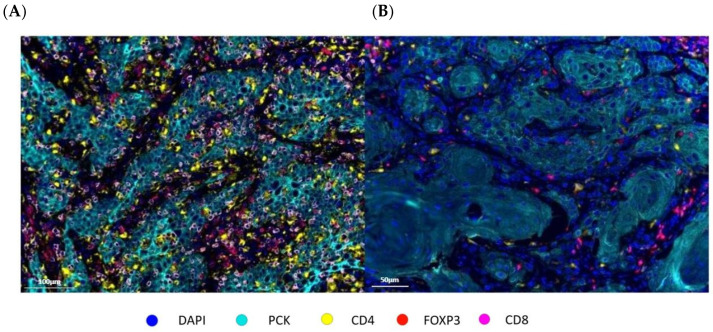
mIF illustration of high versus low cell density and clustering of CD4^+^ Lymphocytes. Representative multiplex immunofluorescence (mIF) images illustrating high (**A**) versus low (**B**) CD4⁺ lymphocyte cell density and spatial clustering within the tumor microenvironment.

**Figure 4:**
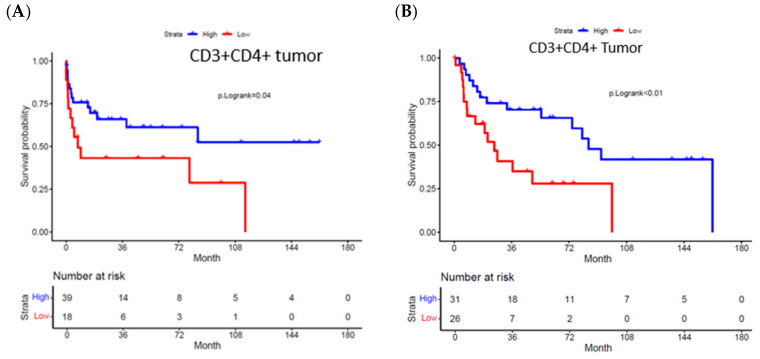
Overall survival (OS) according to helper T cell density and spatial clustering. (**A**) Kaplan–Meier analysis of OS stratified by high versus low intratumoral density of CD3^+^CD4^+^ helper T cells. Median OS was not reached in the high-density group and was 7.4 months in the low-density group. (**B**) Kaplan–Meier analysis of OS based on bivariate intratumoral clustering between CD3^+^CD4^+^ helper T cells and tumor cells. Median OS was 84 months in the high-clustering group and 20.6 months in the low-clustering group.

Figure 3 illustrates mIF images of high versus low cell density and clustering patterns of CD3^+^CD4^+^ helper T lymphocytes within two different patient tumor specimens. High versus low intratumoral density of helper T cells (CD3^+^CD4^+^) were associated with improved OS; not reached versus 7.4 months, *p* = 0.04. HR = 0.38, 95% CI: 0.154–0.938, *p* = 0.036 (Figure 4A). Similarly, high versus low cell density of CD3^+^CD4^+^ helper T cells in the stromal compartment was associated with an OS benefit (84 versus 7.5 months, *p* = 0.024). Bivariate analysis showed close clustering of CD3^+^CD4^+^ cells to tumor cells was associated with a median OS benefit 84 versus 20.6 months, *p* < 0.01. HR = 0.68, 95% CI: 0.485–0.953, *p* = 0.025 (Figure 4B).

**Figure 5:**
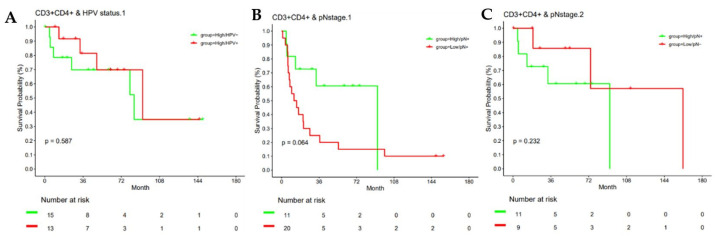
Kaplan–Meier analyses of overall survival according to bivariate intratumoral clustering of CD3^+^CD4^+^ helper T cells across clinical subgroups. (**A**) Overall survival stratified by HPV status among patients with high bivariate intratumoral clustering of CD3^+^CD4^+^ helper T cells. (**B**) Overall survival stratified by nodal status (node-positive vs. node-negative) according to bivariate intratumoral clustering of CD3^+^CD4^+^ helper T cells. (**C**) Overall survival stratified by bivariate intratumoral clustering of CD3^+^CD4^+^ helper T cells in patients with node-positive disease (≥pN1), demonstrating no statistically significant difference between high and low clustering groups.

As expected, survival analysis stratified by HPV status in our cohort showed a median OS benefit in favor of HPV-positive status: HR 2.9, 95% CI (1.1–8.3), *p* = 0.034. However, stratifying by HPV status revealed that overall survival outcomes did not differ in patients with high bivariate clustering of CD3^+^CD4^+^ (Figure 5A). Predictably, node-positive status was associated with a poorer overall survival HR 5.28; 95% CI (2.17–12.84), *p* < 0.001. In patients with node-positive disease, high versus low bivariate clustering of CD3^+^CD4^+^ cells showed a median OS benefit of 94 versus 20 months, although not statistically significant, *p* = 0.064 (Figure 5B). In comparing high bivariate clustering of CD3^+^CD4^+^ in node-positive patients to low bivariate clustering in node-negative patients, there was a lack of statistically significant survival benefit between the two groups, *p* = 0.22 (Figure 5C).

## 4. Discussion

In this study, we employed mIF to profile the immune microenvironment in PSCC. We examined how immune cell densities and spatial clustering metrics within the tumor immune microenvironment are associated with survival outcomes.

In evaluating the TIME, we observed substantial survival outcome differences in the cell density patterns of pro-immunogenic M1 macrophages (CD68^+^CD163^−^CD206^−^). High versus low intratumoral and stromal cell density of M1 macrophages were associated with improved overall survival.

M2 macrophages play a central role in the TIME of other SCC, including head and neck, oral, esophageal, and lung subtypes. These cells are typically identified by markers such as CD163 and CD206 and are distinguished from M1 macrophages by their anti-inflammatory, pro-tumorigenic functions [25,26,27,28]. Similarly, M2 macrophages play a central role in the TIME of PSCC, where they are associated with disease progression, immune suppression and poor prognosis [18,29].

Using single-cell RNA sequencing, Miyagi et al. demonstrated that as PSCC advances from localized to metastatic stages, there is a progressive increase in the density and predominance of M2 macrophages within the tumor and at the invasive margin. This shift is accompanied by a decline in cytotoxic T cell infiltration and a rise in immune exhaustion markers, such as PD-L1, further contributing to an immunosuppressive microenvironment [19]. The presence of M2 macrophages in PSCC is also linked to the suppression of effective anti-tumor immune responses, including the inhibition of cytotoxic T cell activity and the promotion of regulatory T cell recruitment. This immunosuppressive milieu facilitates tumor progression and metastasis.

Importantly, the predominance of M2 macrophages and high PD-L1 expression in advanced PSCC provides a biological rationale for the use of immune checkpoint inhibitors, although clinical benefit is limited to a subset of patients [30]. Mechanistically, M2 macrophages facilitate tumor progression through several pathways. They promote epithelial–mesenchymal transition (EMT) via transforming growth factor beta (TGF-β)/Smad signaling, enhancing metastatic potential and tumor cell proliferation, as demonstrated in lung and laryngeal SCC [28]. M2 macrophages also secrete growth factors such as EGF and VEGF, supporting angiogenesis, tumor cell migration, and invasion [27,29]. In oral SCC, direct contact between M2 macrophages and tumor cells increases migration and invasion, while M1 macrophages exert the opposite effect [31].

The results of our study are consistent with contemporary data with respect to the significance of type 1 versus type 2 macrophages in the TIME of PSCC and provide further evidence for the development of macrophage-directed targeted therapy. We specifically demonstrate that geospatial proximity clustering of M2 macrophages to each other and to tumor cells results in worse overall survival. Importantly, the close spatial association between macrophages and tumor cells observed in our study also has therapeutic implications. The bivariate clustering of M2 macrophages with tumor cells within the intratumoral compartment highlights the intrinsic ability of macrophages to home to and physically interact with malignant cells in PSCC. This spontaneous tumor–macrophage proximity provides a biologic rationale for macrophage-directed cellular therapies, as it suggests that macrophages naturally localize to relevant tumor niches. Therapeutic strategies that exploit this tumor-homing behavior—such as chimeric antigen receptor macrophages (CAR-Ms)—may therefore be well suited to reprogram macrophages within the PSCC tumor immune microenvironment.

Therapeutic strategies targeting M2 macrophages are under investigation. These include depletion of M2 macrophages, reprogramming them toward an M1 macrophages phenotype, and inhibiting key cytokines (e.g., TGF-β, IL-10) or signaling pathways (e.g., JAK/STAT) involved in their polarization and function [25,28,32]. Such approaches may enhance the efficacy of chemotherapy and immunotherapy by reversing immunosuppression and restoring anti-tumor immunity.

Pierini et al. demonstrated that CAR-M targeting HER2 have demonstrated the ability to sensitize HER2-positive solid tumors to PD-1 blockade in preclinical models. In fully immunocompetent syngeneic mouse models, anti-HER2 CAR-M therapy led to significant tumor burden reduction, prolonged survival, and remodeling of the TIME. This remodeling was characterized by increased infiltration of T cells and natural killer (NK) cells, as well as induction of antigen spreading, which contributed to long-term, T cell-dependent anti-tumor immunity [33]. Given the largely disappointing role of immune checkpoint blockage therapy, the addition of macrophage-directed therapy may be a potential future breakthrough in PSCC.

In our analysis of helper T cells within the TIME of PSCC, we noted several interesting findings. Bivariate analysis showed close clustering of CD3^+^CD4^+^ cells to tumor cells was associated with improved median OS. However, stratifying patients by HPV status surprisingly revealed that overall survival outcomes did not differ in patients with high bivariate clustering of CD3^+^CD4^+^, suggesting that the spatial clustering of helper T cells may serve as an HPV status agnostic biomarker in PSCC.

Helper T cells (CD3^+^ CD4^+^ T cells) play a central role in orchestrating the TIME of PSCC. In penile cancer, their function and abundance are thought to be influenced by both disease stage and HPV status. HPV-positive PSCCs generally exhibit higher overall T cell infiltration, including helper T cells, and a Th1-polarized immune response, which is associated with increased cytotoxic activity and immune activation. On the other hand, HPV-positive tumors also show increased regulatory T cell infiltration, which may contribute to immune evasion [34,35]. It has been shown that helper T cells are more abundant and functionally active in early-stage and HPV-positive PSCC, but their numbers and efficacy decline with advancing disease and in HPV-negative tumors, contributing to immune escape and tumor progression [36,37].

In our study, we did not observe a difference in cell density of T lymphocytes by HPV status, and moreover, we noted that high clustering of helper T cells to tumor cells conferred a similar survival regardless of HPV status, suggesting there is no difference in the functionality of helper T cells based on HPV status. These findings have been echoed by Kortekaas et al. in their study of vulvar squamous cell carcinoma, a solid tumor also associated with HPV infection. The authors showed that high numbers of activated helper T cells (specifically, intraepithelial CD4^+^ T cells with an activated phenotype) are associated with better clinical outcomes in early-stage vulvar squamous cell carcinoma (VSCC), regardless of HPV or p53 status. This association was reflected in longer recurrence-free and overall survival. The beneficial prognostic impact of these T cells was independent of the molecular subtype, meaning it applies to HPV-driven, HPV-negative/p53 wild-type, and HPV-negative/p53-mutant VSCCs alike. Tumors with high intraepithelial infiltration by activated helper T cells demonstrate a more robust anti-tumor immune response, which is thought to underline the improved outcomes observed in these patients [38].

Collectively, these results suggest that the immune microenvironment, particularly the presence of activated helper T cells, may be a more powerful predictor of prognosis than HPV or p53 status alone, and our study demonstrates that this may also hold true for PSCC. Single cell RNA sequencing (scRNAseq) has provided valuable insights into the cellular origins of PSCC. A landmark study by Elst et al. established the first scRNA-seq atlas for this malignancy, revealing TP53 mutations as a major driver of aggressive tumor behavior, independent of HPV status [39].

Predictably, node-positive status was associated with a poorer overall survival in our overall population, compared to patients with node-negative disease, HR 5.28; 95% CI (2.17–12.84), *p* < 0.001. We differentiated high versus low bivariate clustering of CD3^+^ CD4^+^ by node-positive status. In patients with node-positive disease, high versus low bivariate clustering of CD3^+^CD4^+^ cells showed improved median OS 94 vs. 20 months, although not statistically significant, *p* = 0.064. Another surprising finding was discovered in comparing high bivariate clustering of CD3^+^CD4^+^ in node-positive patients to low bivariate clustering in node-negative patients. We found there was a lack of statistically significant median overall survival benefit between the two groups, *p* = 0.22. The absence of statistically significant differences in survival between node-negative and node-positive disease based on differential clustering patterns of CD3^+^CD4^+^ T cells, highlights the importance of tumor-infiltrating CD4^+^ lymphocytes in PSCC. The prognostic significance of helper T cells in PSCC is underscored by studies linking higher densities of tumor-infiltrating CD4^+^ T cells to improved disease-specific survival, while increased Treg infiltration and immune checkpoint expression correlate with poorer outcomes [40]. Our study, like others, highlights the role of T helper cells as potential therapeutic targets or biomarkers for immunotherapy response [41,42].

Advancements in high-throughput imaging technologies have brought spatial modeling methods to the forefront of cancer research. mIF enables detailed analysis of tumor–immune interactions at single-cell resolution using customizable antibody panels, tailored to the cancer type or specific clinical objectives. These markers facilitate the identification of immune and tumor cell phenotypes and allow for the assessment of cell-to-cell interactions at both global and localized spatial levels.

To our knowledge, this is the first study to compare survival outcomes in PSCC by cell density and spatial clustering patterns of various immune effector cells using mIF. A major strength of our study lies in the use of mIF, an advanced quantitative method that allows for the simultaneous detection of multiple biomarkers within a single tissue section. This approach enabled a detailed characterization of cellular composition, cell density, and spatial distributions in a comparatively large cohort of patients with this rare malignancy.

The limitations of this study are the evaluation of PD-L1 expression at the TMA level, rather than at the cellular level. This distinctly limited our ability to study and understand other patterns of immune exhaustion within the TIME of PSCC. We also note that the tissue samples included in this study all represent primary tumor resections, limiting our ability to study cellular changes with respect to treatment specific signatures within the TIME of resected nodal tissue. An additional limitation of this study is the limited racial diversity of the cohort, with small numbers of African American and Asian patients. These sample sizes were insufficient to support adequately powered race-stratified analyses.

Future efforts will focus on complementing mIF capabilities with a combination of high-throughput technologies such as spatial transcriptomics, single cell sequencing, proteomics, whole genome sequencing, and circulating tumor DNA (ctDNA) to further map the TIME, identify spatial patterns of immune cell infiltration, track cancer progression and heterogeneity, and guide future therapeutic strategies.

## 5. Conclusions

In this study, we investigated the prognostic value of cell densities and spatial clustering patterns of various immune effector cells within the immune microenvironment of PSCC using mIF. We show that high cell densities of M1 macrophages within the tumor compartment were associated with improved OS, whereas a high clustering of M2 macrophages to tumor cells within the tumor compartment conferred significantly worse outcomes. Within the stromal compartment, a high clustering of M2 macrophages to one another is also associated with a significantly worse overall survival. Our study shows that a high tumor infiltration and clustering pattern of helper T lymphocytes is associated with improved overall survival regardless of HPV status or node-positive status. These findings offer a critical insight into potential therapeutic development and future areas of focus for immune escape mechanisms in penile cancer. This study is limited by its relatively small cohort size and limited racial diversity, reflecting the rarity of PSCC and the demographics of the institutional catchment area.

## Figures and Tables

**Table 1:** Patient Characteristics.

Characteristic (*n* = 57)	N (%)
Age at diagnosis (y), median (IQR)	60 (51–76)
Race	
Caucasian	44 (77)
Hispanic	9 (15.8)
African American	3 (5.3)
Other	1 (1.8)
Pathologic Grade	
Grade 1	38 (66.7)
Grade 2	16 (28.1)
Grade 3	3 (5.3)
HPV status (ISH)	
HPV −	34 (59.6)
HPV +	23 (40.4)
Pathologic T staging	
pT1	17 (29.8)
pT2	16 (28.1)
pT3	22 (38.6)
pT4	2 (3.5)
Pathologic N stage	
pN0	26 (45.6)
pN1	6 (10.5)
pN2	20 (35.1)
pN3	5 (8.8)

**Table 2:** Survival and oncological outcomes.

Variable		N = 57	%
Recurrence	Yes	26	45.6
	No	31	54.4
Recurrence type	Local	5	8.8
	Regional	15	26.3
	Distant	6	10.5
Vital status	Alive	26	45.6
	Dead from disease	21	36.8
	Died of other	10	17.5
Adjuvant therapy	Yes	26	45.6
	No	21	36.8
Time to recurrence	6.3 (3.1–12.7)		
Median OS	36.3 (18.9–52.31)		
Median follow up	45.5 (29.3–93.6)		

**Table 3:** Univariate cox proportional modelling for OS.

Covariate		N	HR (95% CI)	*p* Value
Race	Non-white	13	0.76 (0.26–2.26)	0.62
	White	44	−	
pTstage	1	17	−	0.302
	2	16	1.7 (0.7–4.46)	
	3–4	24	2.08 (0.79–5.48)	
pTgrade	G1	38	−	
	G2–3	19	1.75 (0.84–3.65)	0.134
LVI	Yes	32	1.32 (0.62–2.8)	0.469
	No	23		
HPV (ISH)	+	23	0.34 (0.12–0.92)	0.034
	−	34	−	
PDL1 status	+	33	1.23 (0.78–3.39)	0.127
	−	24	−	
pNstage	2–3	25	7.70 (2.58–22.96)	<0.001
	0–1	32		
Adjuvant therapy	Yes	17	1.77 (0.82–3.82)	0.146
	No	40		

**Table 4:** Phenotypic distribution by cell density across P1 and P2 mIF panels.

Marker Co-Expression	Phenotype	Median Cell Density Total (Cell/mm^2^)	Median Cell Density Tumor	Median Cell Density Stroma
CD68^+^	Total macrophage	322.95	253.94	428.88
CD68^+^CD163^−^CD206^−^	M1 Macrophage	157.64	128.03	170.88
CD68^+^CD163^+^	M2 Macrophage	58.79	45.3	92.9
CD68^+^CD206^+^	M2 Macrophage	48.09	31.27	65.24
CD20^+^	B cell activation	4.82	2.13	7.81
NKp46^+^	Natural Killer	6.7	4.59	7.99
CD3^+^	Total T cells	45.45	27.96	103.16
CD3^+^CD8^+^	Cytotoxic T cells	10.11	7.28	15.7
CD3^+^CD4^+^	Helper T cells	12.2	4.28	33.36
FOXP3^+^	Regulatory T cells	12.19	7.58	36.19
CD45RO^+^	Memory T cells	519.24	295.92	782.21

## Data Availability

The data presented in this study are available on request from the corresponding author.
